# Long-Term Functional Dynamics of an Aphidophagous Coccinellid Community Remain Unchanged despite Repeated Invasions

**DOI:** 10.1371/journal.pone.0083407

**Published:** 2013-12-13

**Authors:** Christine A. Bahlai, Manuel Colunga-Garcia, Stuart H. Gage, Douglas A. Landis

**Affiliations:** 1 Department of Entomology, Michigan State University, East Lansing, Michigan, United States of America; 2 Center for Global Change and Earth Observations, Michigan State University, East Lansing, Michigan, United States of America; 3 Global Observatory for Ecosystem Services, Michigan State University, East Lansing, Michigan, United States of America; Centro de Investigación y de Estudios Avanzados, Mexico

## Abstract

Aphidophagous coccinellids (ladybeetles) are important providers of herbivore suppression ecosystem services. In the last 30 years, the invasion of exotic coccinellid species, coupled with observed declines in native species, has led to considerable interest in the community dynamics and ecosystem function of this guild. Here we examined a 24-year dataset of coccinellid communities in nine habitats in southwestern Michigan for changes in community function in response to invasion. Specifically we analyzed their temporal population dynamics and species diversity, and we modeled the community’s potential to suppress pests. Abundance of coccinellids varied widely between 1989 and 2012 and became increasingly exotic-dominated. More than 71% of 57,813 adult coccinellids captured over the 24-year study were exotic species. Shannon diversity increased slightly over time, but herbivore suppression potential of the community remained roughly constant over the course of the study. However, both Shannon diversity and herbivore suppression potential due to native species declined over time in all habitats. The relationship between Shannon diversity and herbivore suppression potential varied with habitat type: a positive relationship in forest and perennial habitats, but was uncorrelated in annual habitats. This trend may have been because annual habitats were dominated by a few, highly voracious exotic species. Our results indicated that although the composition of the coccinellid community in southwestern Michigan has changed dramatically in the past several decades, its function has remained relatively unchanged in both agricultural and natural habitats. While this is encouraging from the perspective of pest management, it should be noted that losses of one of the dominant exotic coccinellids could result in a rapid decline in pest suppression services if the remaining community is unable to respond.

## Introduction

Aphidophagous coccinellids (ladybeetles, order Coleoptera) are a well-studied group of insects [1,2]. Coccinellids are economically important for their ability to suppress pest herbivores, and their ubiquity and charismatic appearance has led to public interest in their conservation [1]. Over the last 30 years the addition of multiple exotic species to the North American coccinellid community, coupled with observed declines in native species, has led to renewed interest in the ecosystem function of this guild [2]. 

Aphidophagous coccinellids have been under systemic surveillance at the Long Term Ecological Research (LTER) site at Kellogg Biological Station since 1989. This community has been invaded four times in the last 28 years. *Coccinella septempunctata*, a European species [3], arrived in 1985; *Harmonia axyridis*, an Asian species [4], was first detected in 1994; *Hippodamia variegata*, a Eurasian species [5], was first captured in 1999; and *Propylea quatuordecimpunctata*, a European species [6], has been in the region since 2007. 

Prior studies have shown that coexisting coccinellid species partition habitat use in space and time and thus function complimentarily [7]. This allows for more effective resource partitioning and less direct competition between predators [8]. Thus, in a situation with limited competition between native and exotic species, herbivore suppression services offered by a coccinellid community are expected to increase with biodiversity, that is, the addition of exotics may improve herbivore suppression services, provided native species are not extirpated. Moreover, exotic species would be expected to have negligible effect on the diversity of a community if the exotic species effectively replaced a native species with respect to its level of dominance. However, exotic species which are able to outcompete native species are not necessarily superior biological control agents [9,10]. Superior competitors may instead out-compete native species by interfering with the ability of the native species to acquire resources [11]. It has not yet been demonstrated how exotic species affect ecosystem services provided by the coccinellid community (i.e. aphidophagy), over the long term [2], although several studies have documented declines in particular aphid populations in the short term following the establishment of exotic coccinellids [12-14]. From a theoretical perspective, it is expected that exotic species that use resources differently or have greater phylogenetic distinctiveness would have larger impacts on ecosystem functions [15,16], and thus, in the case of coccinellid invasions in landscapes that are already well exploited by native coccinellids, the impact on community function would be expected to be minimal. 

In order to estimate the changes in the ability of the coccinellid community to suppress herbivores before and after invasion, a reliable metric must be used. Bahlai et al. [17] developed the natural enemy unit (NEU), a metric for quantifying the herbivore suppression potential of a resident natural enemy community based on the voracity and abundance of individual species. This metric provides a tool to estimate the predation capacity of the coccinellid community, with the assumption that competitive interactions do not reduce the overall voracity. Thus, this metric allows herbivore suppression potential (i.e.: the maximum number of prey items a resident natural enemy guild is capable of consuming, under ideal conditions) to be evaluated relative to ecosystem characteristics such as biodiversity, and disturbances like invasions. Although competitive interactions between natural enemies are not explicitly considered by this metric, a model using NEUs to represent the herbivore suppression of diverse natural enemy guild corresponded well to field-observed aphid suppression when it was assumed the herbivore suppression potential was the upper asymptote of a type III functional response [18]. Thus, the metric provides the most realistic estimation of realized herbivore suppression by coccinellids in prey-rich environments.

While numerous prior studies have examined aphidophagous coccinellid communities in North America [2,7,12,13,19-29], almost all of the comprehensive, systemic multiyear studies focus on a single crop; predominantly on cultivated herbaceous host habitats [2]. In contrast, our study documented the coccinellid community in southern Michigan from 1989 to 2012 in nine habitats, representing not only one of the longest durations of continuous coccinellid surveillance, but also presents the most diverse set of habitats monitored in a single study. These data also represent a rare case where detailed observations were taken before, during and after the invasion of several species using a standard protocol [11]. In the present paper, we examine herbivore suppression and diversity function in these invaded coccinellid communities over 24 years. 

## Methods

### Description of study site and classification of habitats

All studies occurred at Michigan State University’s Kellogg Biological Station (KBS) in southwestern Michigan (42°24’N, 85°24’W) at 288 m elevation. Coccinellid surveillance data were collected starting in 1989 at the KBS Long Term Ecological Research (LTER) site as part of the Main Cropping System Experiment (MCSE) and forest sites. See http://lter.kbs.msu.edu/research/long-term-experiments/main-cropping-system-experiment/ and http://lter.kbs.msu.edu/research/long-term-experiments/successional-and-forest-sites/ for a detailed experimental design. The MCSE consists of seven treatments including treatments of annual field crops (maize, soybeans and wheat) in annual rotation under 4 levels of management intensity (conventional, no-till, reduced input, and biologically based), as well as alfalfa, poplar and early successional vegetation (i.e. abandoned agricultural fields). Each treatment is replicated six times with individual plot sizes of 1 ha. Observations are taken from five permanent sampling stations within each treatment-replicate combination. Instead of grouping by management intensity for this analysis, coccinellid abundance data from the MCSE were grouped by the particular host crop planted in that treatment-replicate combination in a given observation-year combination. Forest sampling began in 1993, at sites within 3 km of the MCSE on the KBS. Forest types included: 40-60 year old conifer forest plantations, late successional deciduous forest, and successional forests occurring on abandoned agricultural land. Three 1 ha replicates of each forest type are monitored at five sampling stations, as in the MCSE. The LTER experiment continues to date. Detailed agronomic records for the site are available at http://lter.kbs.msu.edu/datatables/150. Coccinellid abundance data is available online at http://lter.kbs.msu.edu/datatables/67. A relational database in MS Access® integrating coccinellid data with the relevant environmental and agronomic records used in this analysis is available from the authors by request.

To monitor coccinellids, un-baited two-sided yellow cardboard sticky cards (Pherocon, Zoecon, Palo Alto CA) are suspended at 1.2 m above ground at each sampling station [29]. Each week, coccinellids on the sticky cards are identified using a pictorial key, counted, recorded, and then removed from sticky cards. Sticky cards are replaced every two weeks. The length of the sampling period within the growing season averaged 14 weeks (with a standard error of one week, and minimum and a maximum of 8 and 22 weeks respectively). Variation depended on the availability of labor, crop type, and weather.

For analyses and data presentation, individual habitats (i.e. dominant plant community occurring in a treatment block) were grouped into three habitat types: annual crops, perennial crops, and forest habitats. Annual crops were all dominated by annual herbaceous plants, grown in near monoculture, and included maize, wheat, and soybeans. Habitats classed as perennial crops included poplar, alfalfa, and managed early successional. Managed early successional habitats were included in this group because, although this community is not a crop *per se*, it is a community resulting from abandoned crop land, occurring within a crop matrix, and managed to maintain their early successional stage (e.g., yearly spring burnings). Finally, forest habitats included coniferous, deciduous, and successional forest.

### Coccinellid abundance data

The relative abundance of 13 species of aphidophagous coccinellids (Table 1) as measured by sticky trap capture of adults was used in the analysis. Species were included if they were recorded in the surveys, and had been recorded in the literature as consuming aphids. The majority of coccinellids recorded are primarily aphidophagous, but *Coleomegilla maculata*, an omnivorous species that also feeds on pollen [30], and *Chilocorus stigma*, a scale specialist that incidentally feeds on aphids [31], were included in the analysis. Two other coccinellids, *Brachiacantha ursina*, and *Psyllobora vigintimaculata*, are only known to eat scale insects and fungi, respectively [32], and were excluded from the analysis. 

**Table 1:** Species included in analysis, including Latin names, common names, abbreviations used in analysis, origin, proportion of total captures, and observation notes.

**Species**	**Common name**	Origin*	**% total capture**	**Notes**
*Adalia bipunctata*	Two-spotted	Native	<1	
*Coccinella septempunctata*	Seven-spotted	Exotic	41	First observed at site in 1985, prior to initiation of study
*Coleomegilla maculata*	Pink or Spotted	Native	19	
*Chilocorus stigma*	Twice-stabbed	Native	1	
*Coccinella trifasciata*	Three-banded	Native	<1	
*Cycloneda munda*	Polished	Native	4	Sometimes recorded as *Cycloneda* sp., *C. munda* is the only species in this genus known from this locality
*Hippodamia tredecimpuncta*	Thirteen-spotted	Native	<1	Last observed at site in 2002
*Harmonia axyridis*	Multicolored Asian or Harlequin	Exotic	28	First trapped in 1994
*Hippodamia convergens*	Convergent	Native	<1	
*Hippodamia glacialis*	Glacial	Native	<1	First trapped in 1994
*Hippodamia parenthesis*	Parenthesis	Native	2	
*Hippodamia variegata*	Variegated	Exotic	1	First trapped in 1999
*Propylea quatuor-decimpunctata*	Fourteen-spotted	Exotic	1	First trapped in 2007

49].^*^ based on information presented in Gordon [

Data were examined at two different temporal resolutions to gain insight into both real-time interactions and between season trends. First, the sum of all captures from each sampling station in a given week, in a given treatment by replicate combination, was called ‘weekly average,’ and the sum of all captures in a given treatment over a calendar year, was referred to as ‘yearly total.’ Data were further subdivided into sets of ‘all coccinellids’ and ‘native species only’ to detect differences in diversity and function of native coccinellids compared to the community as a whole. To aide in data visualization, a Gaussian smoother with a span of 3 units was used to produce smoothing curves. To reduce the likelihood of erroneous conclusions from a large dataset with numerous sources of variation, α=0.01 was observed for all analyses.

### Diversity analysis

The Shannon Index (Shannon’s H) was used to examine trends in diversity of the coccinellid community at the site over time [33]. A Shannon Index was computed for the species distribution at each observation and then this value was used as the dependent variable in several analyses examining the Shannon Index by each habitat type. One way ANOVA or, if data did not meet assumptions of ANOVA, Kruskal-Wallis one way analysis of variance on ranks were performed using SigmaPlot 11.0.0.75 For Windows (Systat Software Inc.) for Shannon Index by habitat for both datasets. Weighted least square regressions were conducted in R 2.15.3 [34] to detect linear trends in Shannon Indices over the observation period. Regressed data points were weighted by number of traps reporting in a given replicate-week combination, or yearly total number of traps in a habitat, for weekly and yearly datasets, respectively. 

### Herbivore suppression potential model

The herbivore suppression potential of the observed community was modeled by weighting each species by its relative voracity, after Bahlai et al. [17]. Herbivore suppression potential is computed in natural enemy units (NEU), where 1 NEU is the number of predators or parasitoids required to kill 100 of a particular pest insect in 24h. Thus,

$$NEU_{total}=\sum_{i=1}^{N}n_{i}V_{i}$$

for *N* species, where *n*_*i*_ is number of individuals of species *i*, and *V*_*i*_ is the voracity of species *i*, expressed as the number of target prey (in this case, aphids) consumed in 24h, divided by 100. 

### Voracity of adult coccinellids

To estimate the voracity of each coccinellid species, literature values of average aphid consumption rates were used. If a study reported aphid consumption rates for each sex for a given species, a 1:1 sex ratio was assumed and the average of the consumption rates are reported. Where multiple sources were available, the highest estimate of voracity was used, except when the highest estimate was dramatically higher than all other published values; then the top two estimates were averaged. The voracity values used to estimate herbivore suppression potential for all aphidophagous coccinellids examined in this study (obtained from the literature) are provided in Table 2.

**Table 2:** Estimated adult relative voracity for 13 species of aphidophagous coccinellids.

**Coccinellid Species**	**Prey Species**	**Voracity (*V*_*i*_)**	**Reference**
*Adalia bipunctata*	*Myzus persicae*	0.172	Ellingsen [50]
*Coccinella septempunctata*	*Aphis glycines* (82.5), *Diuraphis noxia* (43.1)	0.628	Xue et al. [51], Michels and Flanders [52]
*Coleomegilla maculata*	“prey”, including aphids and mites	0.350	Lucas et al. [53]
*Chilocorus stigma*	Incidental feeding on aphids, scale specialist	~0.000	Muma [31]
*Coccinella trifasciata*	*Acyrthosiphon pisum*	0.320	Yadava and Shaw [54]*
*Cycloneda munda*	*Toxoptera citricida*	0.384	Morales and Burandt [55]**
*Hippodamia tredecimpunctata*	*Diuraphis noxia*	0.189	Michels and Flanders [52]***
*Harmonia axyridis*	*Aphis glycines* (88.5), *Myzus persicae* (45.8)	0.672	Xue et al. [51], Soares et al. [56]
*Hippodamia convergens*	*Acyrthosiphon pisum*	0.440	Yadava and Shaw [54]*
*Hippodamia glacialis*	*Acyrthosiphon pisum*	0.440	Yadava and Shaw [54]* ^,^ ****
*Hippodamia parenthesis*	*Acyrthosiphon pisum*	0.320	Yadava and Shaw [54]*
*Hippodamia variegata*	*Diuraphis noxia*	0.237	Michels and Flanders [52]***
*Propylea quatuordecimpunctata*	*Diuraphis noxia*	0.200	Michels and Flanders [52]***

*C. maculata*, which is much more similar to other published values than the value reported in this paper of 15 aphids per 24 hours when aphid resources are limited. ^*^ In Yadava paper, prey were not presented in excess of what could be consumed by most coccinellids in 24 h, so adult consumption rates in a 3 hour period were multiplied by 4, assuming feeding activity occurred only in daylight and daylight was approximately 12 h , instead of using the net consumption over 24 h reported in the paper. This transformation yields a value of 40 aphids per 24 hours consumption rate for

*C. munda* is closely related to *C. sanguinea*, and is approximately the same size^**^ No voracity data exists for this species, but

^***^ Based on average consumption rate for several strains of a given species of coccinellid, collected from different localities

No voracity data exists for this species, but *H. convergens* is the *Hippodamia* species closest in size to *H. glacialis*, so *H. convergens* voracity is used here.

### Analysis of herbivore suppression services and relationship with biodiversity

To detect linear trends in herbivore suppression potential over the observation period, one way ANOVA or, if data did not meet assumptions of ANOVA, Kruskal-Wallis one way analysis of variance on ranks were performed for herbivore suppression potential by habitat for both datasets. Least square regressions weighted by the number of reporting traps were performed herbivore suppression potential were examined using data at the weekly resolution only. A least-square linear regression analysis was performed comparing the Shannon Index to herbivore suppression potential for captured coccinellids for each habitat type on the weekly average dataset. Year was treated as a factor in the analysis to account for year-to-year variability in overall coccinellid occurrence. ANOVA was performed on the regression model to determine if the interaction of Shannon diversity and herbivore suppression potential differed between habitats.

## Results

### Coccinellid abundance data

Between 1989 and 2012, 739,047 individual observations were recorded (species by trap) in the KBS LTER coccinellid database, 61,377 traps were monitored, and 57,813 aphidophagous coccinellids corresponding to thirteen species were captured.

### Community structure and dynamics

**Figure 1:**
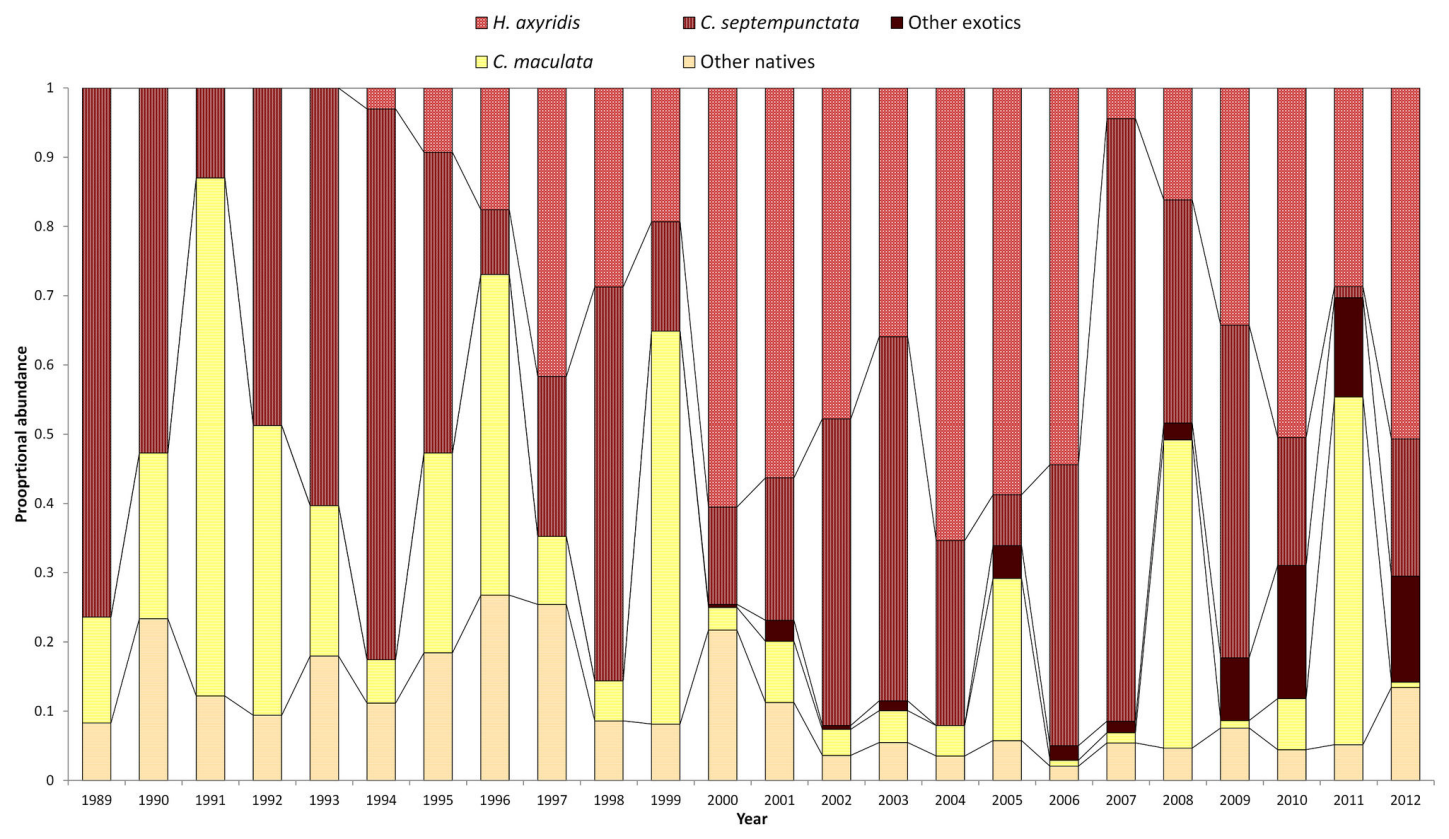
Dominant coccinellid species varies from year to year. Proportional abundance of most common coccinellid species by year. Data were collected at Kellogg Biological Station, Hickory Corners, MI, 1989-2012.

Coccinellid community structure varied markedly over the course of the study (Figure 1). The proportion of total captured coccinellids for each species from 1989 to 2012 is given in Table 1, and the proportional abundance by year of species accounting for ≥1% of total captures is given in Figure 1. Over the entire study, *C. septempunctata*, and more recently, *H. axyridis* comprised the two dominant species, together accounting for more than 69% of total captures. Prior to 1996, *C. maculata* was frequently observed to be co-dominant with *C. septempunctata*, but has declined in importance since, typically only representing greater than 20% of the captures in years when maize is planted at the site. The relative abundance of each of these three dominant species varied from year to year (Figure 1). The two most recent additions to the community, *H. variegata* and *P. quatuordecimpunctata*, are rapidly increasing in dominance. Although absolute proportions of each species captured varied dramatically by year, the community was increasingly dominated by exotic species over the course of the study. Native species represented 49.8% of captures in the first five years of the study. However, between 2008 and 2012, native species represented only 27.9% of the captures, with more than two thirds of these captures corresponding to *C. maculata*.

**Figure 2:**
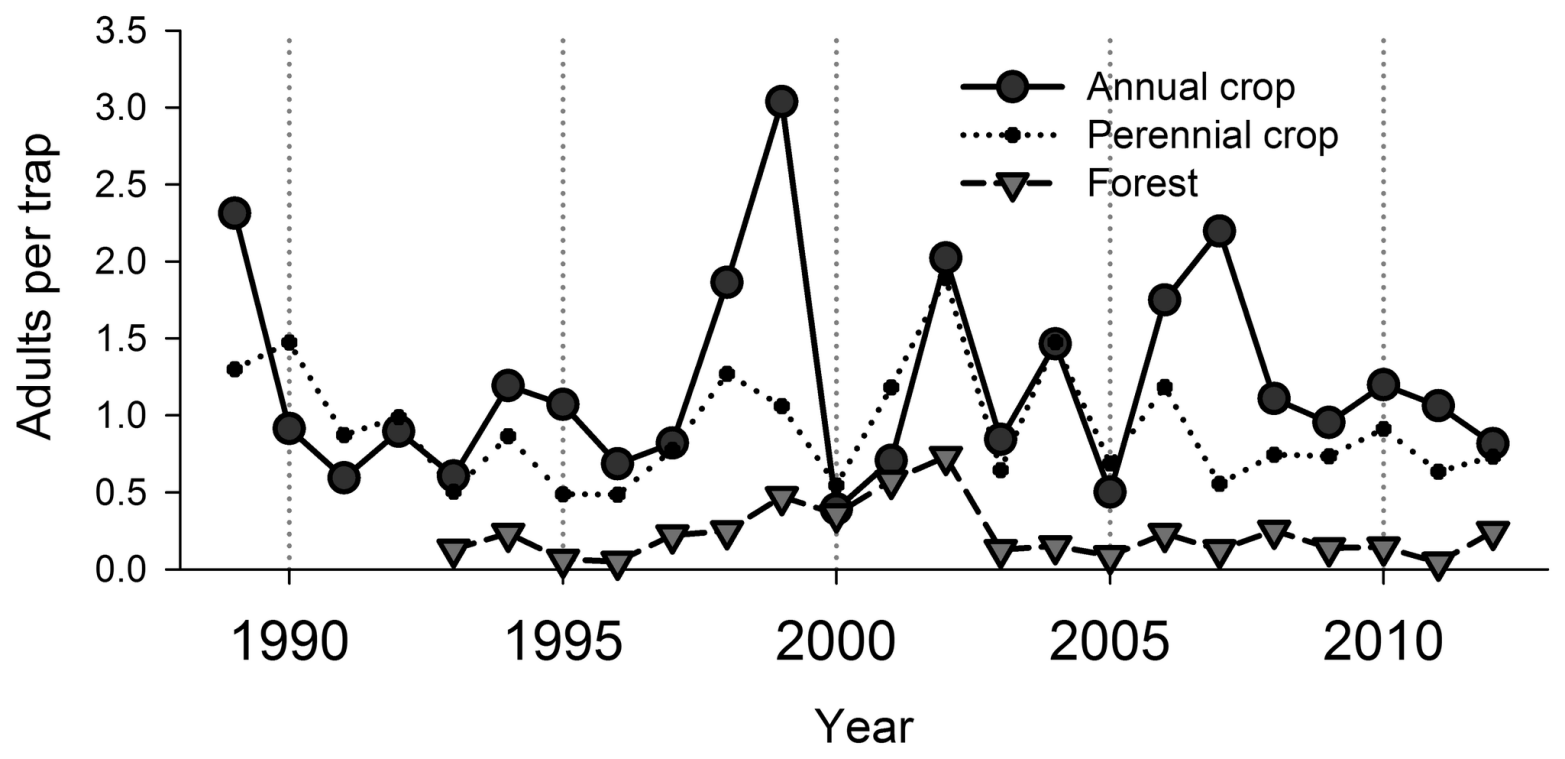
Year-to-year variation in overall coccinellid captures. Average trap captures per year of aphidophagous coccinellids at Kellogg Biological Station, Hickory Corners, MI, 1989-2012, in the annual and perennial crop habitats and in the adjacent forest habitat.

Total coccinellid abundance also varied widely by year over the course of the study (Figure 2). On average, coccinellid captures were highest in annual crops, followed closely by captures in perennial crops, while the trapping rate in forest habitats was consistently lower than that in either annual or perennial crop habitats (Figure 2). 

### Diversity analysis

**Figure 3:**
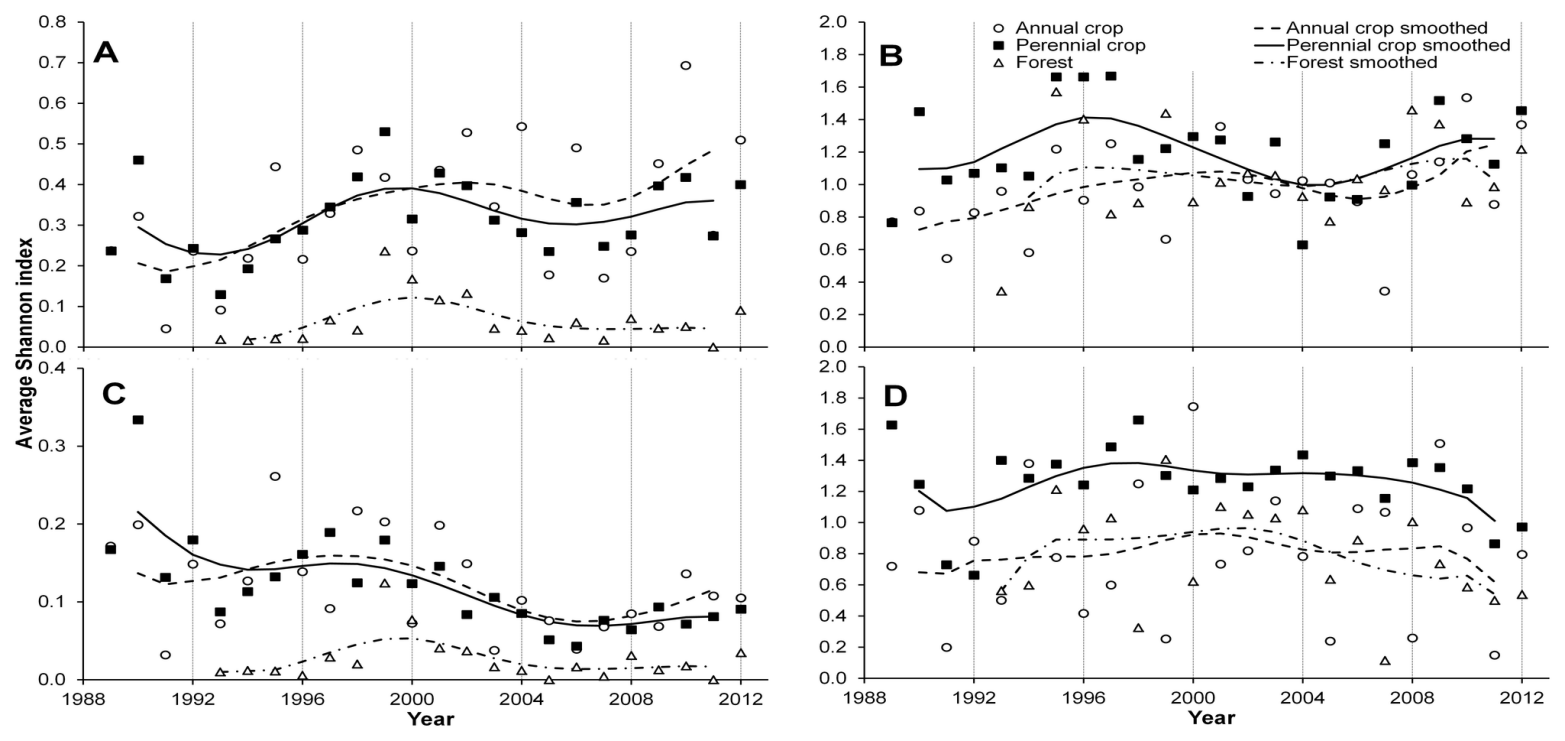
Trends in diversity in the coccinellid community. Shannon index of aphidophagous coccinellid community by habitat type at Kellogg Biological Station, Hickory Corners, MI, 1989-2012, showing observed values (symbols) and 3 yr smoothed (lines) data. **A**) Shannon index analyzed at weekly resolution, all species. **B**) Shannon index analyzed at yearly resolution, all species. **C**) Shannon index analyzed at weekly resolution, native species only. **D**) Shannon index analyzed at yearly resolution, native species.

Shannon diversity varied between habitat types at the weekly resolution, but not at the yearly resolution of the data (weekly: H_2_ =860.7, p<0.01, yearly: H_2_=7.03, p=0.03, Table 3). In a given week, the greatest diversity was observed in annual crops and the lowest diversity was observed in forest habitats. A positive linear trend in Shannon diversity by year was observed at the weekly resolution in annual and perennial crops (annual: slope=0.0102 ±0.0008, p<0.01; perennial: slope=0.0053± 0.0008, p<0.01), but no trends were observed in forest (Figure 3A), nor in Shannon diversity over time within individual habitat groups at the yearly resolution (Figure 3B). When Shannon diversity of only native species was examined at the weekly resolution, a significant linear decrease over time was observed in annual and perennial crop habitats (annual: slope=-0.0031 ±0.0004, p<0.01; perennial: slope=-0.0058± 0.0004, p<0.01), however, no linear relationship was observed in forest habitats (Figure 3C). At a yearly resolution, no significant trends were observed in native coccinellid Shannon diversity (Figure 3D). 

**Table 3:** Analysis of variance* of Shannon’s H at both temporal resolutions (weekly, yearly) and herbivore suppression potential (in natural enemy units) by habitat for thirteen species of aphidophagous coccinellid at Kellogg Biological Station Long Term Ecogical Research site1989-2012.

Habitat type	Shannon's H				Herbivore suppression potential (NEU),	
	Median (Mean), weekly data		Median (Mean), yearly data		Median (Mean), per trap per week	
*All species*						
Annual	0.00 (0.34)	a	0.97 (0.97)	a	0.4 (0.6)	a
Perennial	0.00 (0.32)	a	1.18 (1.20)	a	0.3 (0.5)	b
Forest	0.00 (0.07)	b	1.01 (1.09)	a	0.0 (0.1)	c
*Native species only*						
Annual	0.00 (0.12)	a	0.79 (0.81)	a	0.0 (0.1)	a
Perennial	0.00 (0.12)	a	1.29 (1.25)	b	0.0 (0.1)	b
Forest	0.00 (0.03)	b	0.81 (0.80)	a	0.0 (0.0)	c

^*^ Data failed assumptions of ANOVA for all comparisons except for Shannon’s H for all species at the yearly resolution; Kruskal-Wallis ANOVA on ranks was used. Treatment groupings indicated by letters following median values computed by Dunn's method for multiple comparisons. α=0.01

### Herbivore suppression services and relationship with biodiversity

Herbivore suppression potential of the full coccinellid community varied between habitats (Kruskal-Wallis, H_2_=1610.6, p<0.01, Table 3). Herbivore suppression potential was highest in annual crops, and lowest in forests.

**Figure 4:**
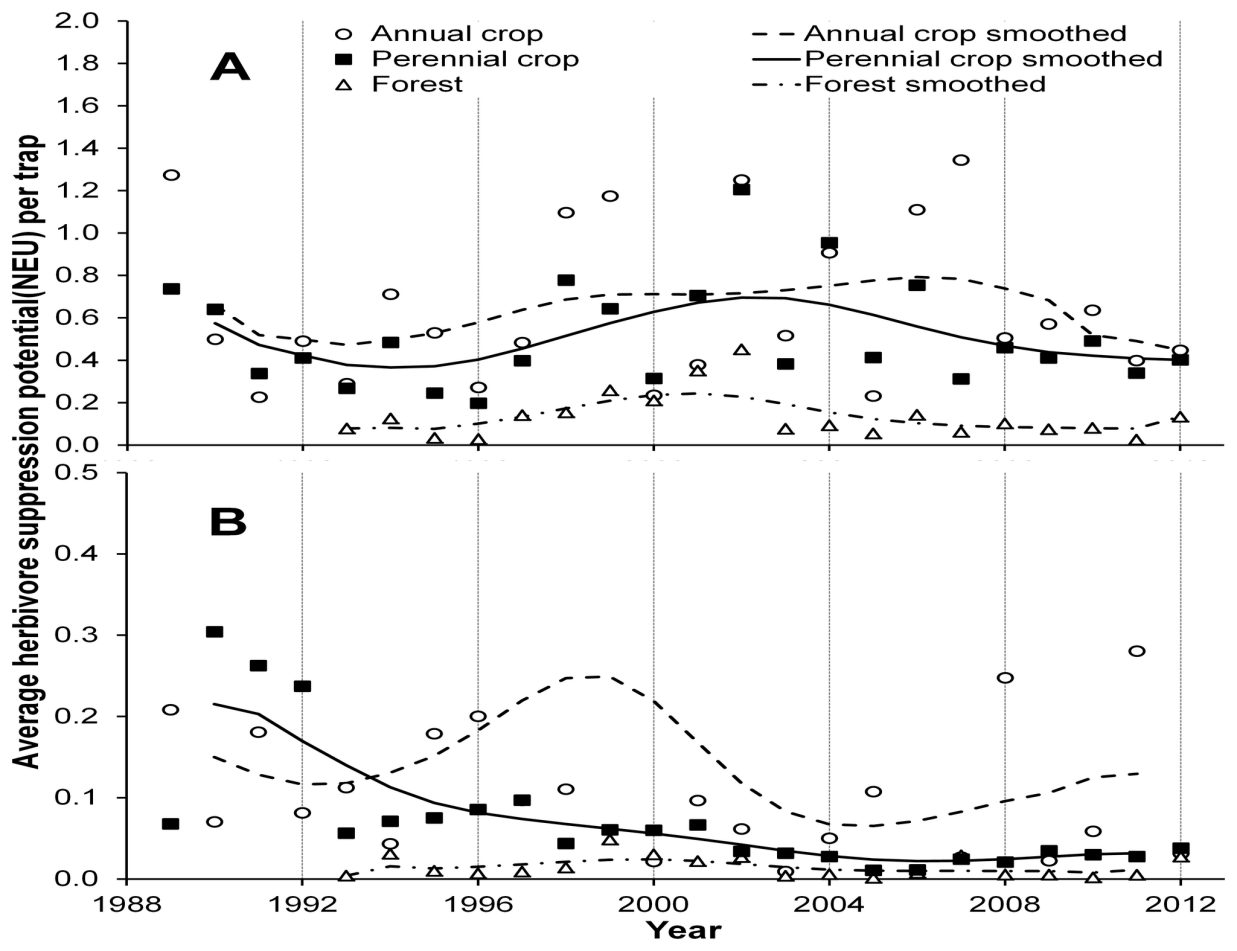
Trends in herbivore suppression potential of the coccinellid community. Herbivore suppression potential (in natural enemy units, NEU) of aphidophagous coccinellid community by habitat type at Kellogg Biological Station, Hickory Corners, MI, 1989-2012, showing observed values (symbols) and 3 yr smoothed (lines) data. **A**) Herbivore suppression potential of all species**. B**) Herbivore suppression potential due to native species only. Note that the scale of the chart excludes one outlier: a value of 0.95 NEU in annual crops in 1999.

No significant trend was observed in the modeled herbivore suppression potential of the coccinellid community over time in any of the habitat groups (Figure 4A). Herbivore suppression potential due to native species exhibited a significant linear decrease over time in annual and perennial crop habitats (annual: slope=-0.0030 ±0.0007, p<0.01; perennial: slope=-0.0074± 0.0005, p<0.01) but no trend was observed in forest habitats (Figure 4B). 

**Figure 5:**
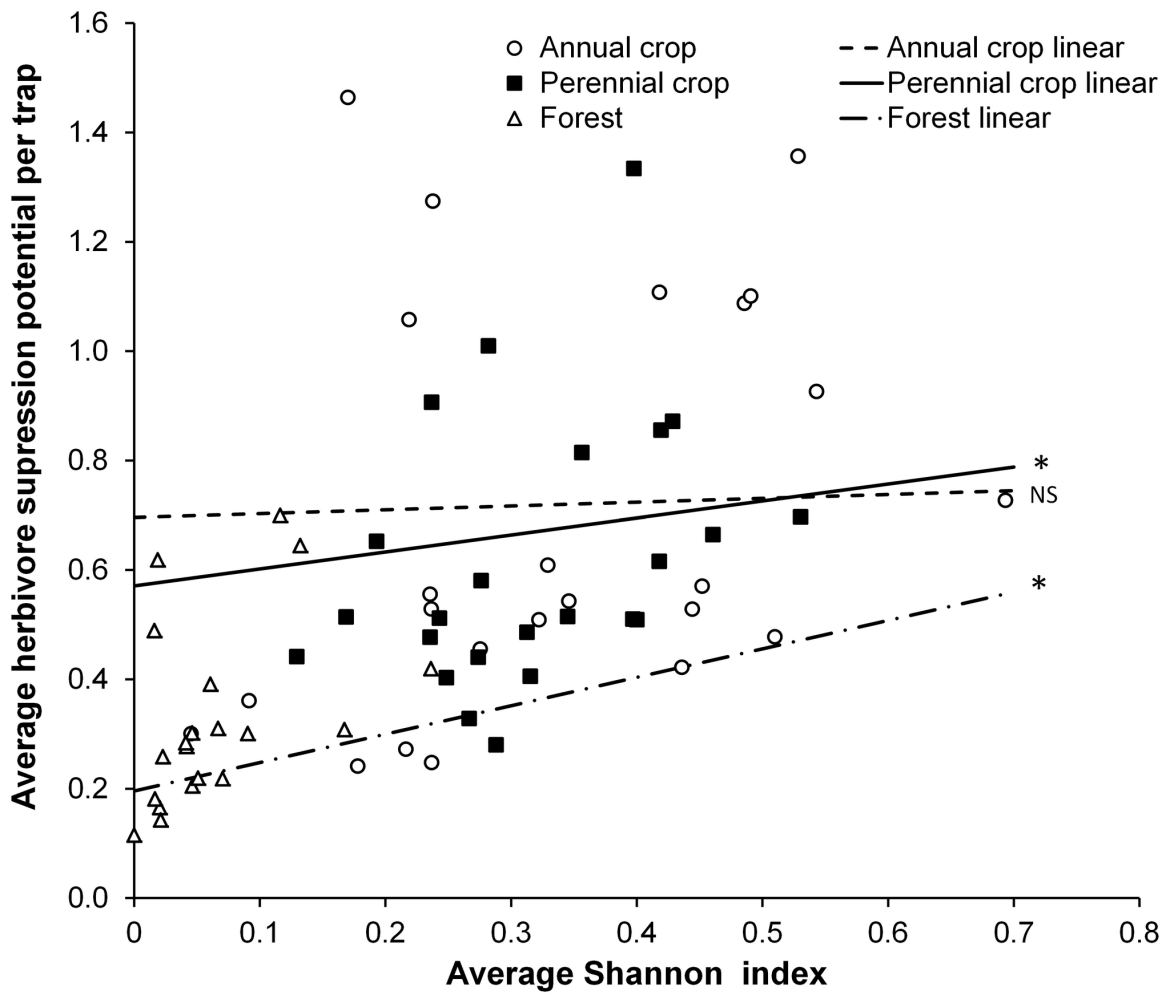
Herbivore potential varies with diversity, except in annual crops. Herbivore suppression potential as a function of Shannon diversity index in three habitat types. For clarity, plotted points represent the average herbivore suppression potential per trap per year vs. the average Shannon index for each repetition by a crop type by sampling week combination in a given year. Regression lines are based on weighted regression of weekly observations. Regression lines marked with an asterisk indicate significant slopes at α=0.01.

Herbivore suppression potential was examined as a function of Shannon diversity index in three habitat types (Figure 5). The relationship between herbivore suppression potential and Shannon diversity varied between habitats (F _2, 9374_ = 24.2, p<0.01); the relationship was positive in perennial and forest habitats (slope =0.24±0.4, 0.47±0.9, respectively) but no significant relationship was found in annual habitats.

## Discussion

### Coccinellid community dynamics

The pattern of long-term co-dominance between several species observed in this study is common in coccinellid communities (i.e., most are dominated by two to four species) although the composition of the group of dominant species may vary with location and time [35]. This co-dominance pattern is not dependent on the invasion or disturbance status of the guild. Similar co-dominance patterns have been observed in a diversity of pre-invasion coccinellid communities [19,22,24,36,37], as well as post-invasion communities [13,25].

Certain coccinellid species tend to dominate captures in areas where they are established. In our study, for instance, *C. septempunctata*, *H. axyridis* and *C. maculata* were among the dominant species in the coccinellid communities when they were present in the community [2,14,23,36,38,39]. *P. quatuordecimpunctata* has been increasing in its relative abundance since its arrival in the system, and it may join the aforementioned co-dominant species, as it, too, is known to co-dominate the coccinellid community in its native range [40].

### Biodiversity and herbivore suppression potential

Net biodiversity of the coccinellid community changed very little over the course of our study. In annual and perennial crop habitats, we observed a small increase in weekly, but not yearly Shannon diversity despite the introduction of three exotic species over the course of the study (Figure 3A, B). Diversity of natives declined in annual and perennial crop habitats at a weekly resolution, and no net change to native diversity was observed over the course of this study at the yearly resolution. This suggests that although native species are still present and just as likely to be captured over the course of a year, they are less likely to be captured within a given week. This result is similar to what has been observed in previous studies. In a review by Harmon et al. [2] combining the observations of several North American coccinellid invasions, the number of native species in an assemblage did not change in response to the addition of an exotic species (i.e., it was rare to observe a complete elimination of a native species associated with the introduction of an exotic species). The diversity index associated with native species only decreased slightly, on average, after the establishment of an exotic species [2].

Modeled herbivore suppression potential of the community also changed very little in response to invasion (Figure 4). Acorn [41] suggested that the interpretation of coccinellid invasion data has been influenced by attitudes against ‘biological pollution’ even though ecological change (of the kind resulting from native species being displaced by exotics) does not yield a loss of function. Our data support this assertion, at least from the standpoint of preserving the function of aphidophagy. We estimated very little change in the long term herbivore suppression potential across our experimental landscape over the course of the study (Figure 4A). However, there was a period of increased variability in herbivore suppression potential immediately after the introduction of soybean aphid, *Aphis glycines*, in 2000. This introduction is known to change the resource structure for coccinellids at landscape scales [42]. During the acute invasion phase of this aphid from 2000 to 2006, coccinellid densities reached higher peaks, and were even more variable than what is typically observed (Figure 2). At this time, a brief depression in overall diversity of the coccinellid community was also observed (Figure 3B), as a single exotic species (*H. axyridis*) became hyper-dominant. Soybean aphid is a preferred prey item of *H. axyridis* and the arrival of this aphid biotically facilitated the invasion of this exotic coccinellid [43]. Soybean aphid outbreaks have since decreased in frequency, and there is evidence that, since 2007, this prey item is no longer acting as a significant driver of population dynamics of *H. axyridis* [44]. 

Diversity was significantly and positively related to herbivore suppression potential in perennial and forest habitats, but was uncorrelated in annual crops (Figure 5). This observation has important implications for biological control in these systems. When coccinellid species are partitioned into spatiotemporal niches, intraspecific competition will be more important than interspecific competition in the population dynamics of a species, intraguild predation will be uncommon, and prey suppression will increase with diversity of the community [7]. The exception to this may be in landscapes with less plant diversity, such as annual cropping systems, which provide a lower diversity of habitats and, in turn, prey species. In this case, a coccinellid species particularly adapted to the particular prey resource available may essentially dominate the entire habitat, to the exclusion of other species. However, functional diversity, that is, the partitioning of resources between consumers with different resource use patterns, is more important than simple biodiversity in determining the level of resource exploitation when resources themselves are diverse [45]. 

### Sampling methodology

Fewer coccinellids were collected in forest habitats than in perennial or annual habitats over the duration of the study. This might be due, at least in part, to decreased efficiency of our trapping design when located in arboreal habitats (see methods). Nevertheless, more coccinellids were captured in managed early successional habitats (within the Main Cropping System Experiment) than in successional forests. The fact that these two types of successional habitats were structurally similar may indicate that indeed fewer coccinellids were present in forest habitats. Although yellow sticky cards are imperfect estimators of relative abundance of coccinellids [46], they represent a cost effective, consistent sampling unit. This characteristic makes them a useful methodology when employed in long term studies, which may be otherwise biased by personnel turnover or inconsistent sampling. Also, the fact they are permanently placed in the field may increase the probability of capturing rare species as they move through the landscape. It is important to note that between habitat and between species comparisons may be affected by the varying trap efficiency for a given species in a given habitat, and thus the strongest evidence for ecological change results from within species, within habitat trends. 

## Conclusions

Despite repeated invasions and a dramatic change in overall composition of the coccinellid community in southwestern Michigan, we find little evidence to suggest a change in function of aphidophagy. Our estimates of biodiversity and herbivore suppression potential, and the overall population dynamics of the community as a whole suggest that this community is currently functioning much as it was at the initiation of this study, and to other similar systems [13,19,22,24,25,36,37].

Long term studies of invaded systems are essential because the impact of exotic species may change over time. Our study confirms prior observations that exotic coccinellids negatively impact community diversity during the acute phases of their invasion (i.e., when they often singly dominate the coccinellid community for several years) [2,47]. Afterwards, the community diversity returns to a ‘new normal’ with several co-dominant species which variably alternate in relative abundance. This variation depends on opportunistic responses to environmental conditions, but is also likely influenced by a given species’ characteristic population fluctuations.

Non-native species that are superior competitors are known to negatively affect the diversity and abundance of closely related or functionally similar native species. However, the direct impact of exotic species on ecosystem functions is more variable depending on the degree of functional overlap and phylogenetic relatedness between the exotic species and the native species that are displaced [11,15]. In guilds of functionally similar predators, capacity for herbivore consumption is controlled by the availability of prey, and is less dependent on the identity of the members of the guild [25]. This does not mean that the loss of native coccinellid biodiversity is without concern, however. Although a low diversity of exotic species may provide equivalent service, native coccinellids provide resilience of herbivore suppression services in a landscape: when prey populations in agricultural systems escape control by exotics, native species quickly colonize pest outbreak areas [14]. Members of insect communities with large body sizes, like the two dominant exotic species at our study site, tend to be among the most functionally efficient members of the community (Table 2), and may also be more prone to local extinction [48]. If native coccinellids continue to decline, and any of the dominant exotic coccinellids which are now very important providers of herbivore suppression service were lost, major disruption to the pest suppression services offered by a landscape could be observed. 
